# Combining the Finite Element Analysis and Kriging Model for Study on Laser Surface Hardening Parameters of Pitch Bearing Raceway

**DOI:** 10.3390/ma15072681

**Published:** 2022-04-06

**Authors:** Hongwei Zhang, Meng Zhu, Siqi Ji, Yantao Dou

**Affiliations:** School of Mechanical Engineering, Beijing Institute of Petrochemical Technology, Beijing 102617, China; zhumeng2048@163.com (M.Z.); jisiqi@bipt.edu.cn (S.J.); douyantao@bipt.edu.cn (Y.D.)

**Keywords:** Kriging model, laser surface hardening, numerical simulation, depth of hardened layer, pitch bearing raceway

## Abstract

Laser surface hardening is used to improve the fatigue performance of the large diameter pitch bearing. Determination of the process parameters by a trial and error method, depending on the experience of the technician, by changing the parameters repeatedly for each laser surface hardening process is time-consuming and costly. In this paper, a method of analyzing the maximum temperature and depth of a hardened layer during the laser surface hardening process for a pitch bearing raceway of a wind turbine is proposed, which combines finite element simulation and the Kriging model. A three-dimensional finite element model of a pitch bearing ring was established using ABAQUS. The temperature field analysis was performed. The effects of process parameters including laser power, scanning speed, and laser spot radius on the depth of the hardening layer were investigated. Then, taking into account the interactional effects of different process parameters, Kriging models were constructed to reflect the relationship between input process parameters and output responses. The results show that the Kriging approximation model has a small relative error compared with the simulated results and can be used to predict the hardened layer depth.

## 1. Introduction

Pitch bearing is an important part of a wind turbine that is installed between the blade and the hub, and the windward angle of the blade are changed from the pitch bearing to obtain the aerodynamic torque and keep the power output stable. The research shows that the working condition of pitch bearing is the worst and the force is the most complex which makes the expected service life difficult to achieve. The pitch bearing has some failure phenomena, such as raceway wear, contact fatigue, outer ring fracture, and so on [1,2,3]. These failures are related to the quality of the surface or subsurface, so the slewing bearing has a great demand for high-quality surface strengthening. Many researchers [4,5,6,7,8] studied the fatigue life considering the hardened layer for the slewing bearing. Their works demonstrated that the hardened layer can affect the service life and the carrying capacity.

Laser surface hardening (LSH) has been widely used for various applications to increase fatigue strength under certain conditions [9,10,11,12,13,14]. The two important parameters to ensure the laser quenching effect are surface temperature and the depth of the hardened layer, which were obtained by adjusting the pre-conditions such as laser power, laser scanning speed, and spot size before exactly the same application is exposed to the actual machine parts.

At present, many studies have researched the effect of laser surface hardening by using experimental and numerical simulation methods [15,16,17,18,19,20,21,22]. Tobar et al. proposed a three-dimensional numerical model of a laser surface treatment combined with experiments to predict the distribution of hardened layer depth [15]. The parameters of the hardening of stainless steel AISI H13 according to this method are optimized. Erica Liverani et al. [16] studied the influence of laser incident flowed rate and scanning speed on the hardening depth and residual stress state of the same component by numerical simulation. Studies have shown that the hardened area is determined by the peak temperature and the temperature interaction time. Moradi et al. [18] performed the experimental tests of laser surface hardening of AISI 420 steel based on the design of an experiment method. The hardened depth and width, the maximum micro-hardness, and the hardness deviation from the base metal were investigated considering the parameters of laser power, laser scanning speed, and focal plane position of the laser beam. Mosavi et al. [22] developed a mathematical model to calculate the temperature distribution on the surface and bulk of a steel plate under the laser hardening process. The model is mainly concerned with the parameters of the laser hardening process: velocity of the laser spot and irradiation time. 

However, the majority of numerical simulation works focused on the reliability of predicted results after LSH treatment, such as verification of temperature, residual stresses, surface roughness, and so on. In the actual process, each parameter changes in a certain range, and the process parameters have a certain correlation and mutual coupling influence. Because of the complex interaction of different process parameters, this can be time-consuming and costly depending on the technician’s technical expertise. Therefore, the design of experiments (DOE) has been developed in various experimental works. In order to reduce the number of experiments, taking into account the interactional effects and developing mathematical functions to achieve the logical relationship between input and output parameters, the response surface method (RSM) has been conducted by many researchers [23,24,25,26,27,28]. On the one hand, with the continuous improvement of the simulation optimization method, the simulation optimization work of the LSH process has gradually increased, including the application of the genetic algorithm, the RSM, and other methods to optimize LSH parameters. A large number of repetitive experiments and precise control of the laser hardening parameters are required. On the other hand, the research goal was only just to obtain the maximum ideal value but without a discussion about the relationship between the results and LSH parameters. Furthermore, there are very few LSH works involving large-scale bearing rings and many are mainly focused on certain materials without considering the structural effect. Moradi et al. [23] studied the laser surface hardening of AISI 410 based on RSM. Moreover, the effects of input parameters on the response variations were investigated by statistical investigation.

The Kriging method is an interpolation method that predicts unknown points of known points. The Kriging method uses variance changes to calculate spatial changes, and the error of the predicted value obtained from the spatial distribution is small. At present, the Kriging method is applied for many fields [29,30,31,32]. Compared with the experiments investigating one factor at a time while keeping other factors constant, the approximating model allows the input parameters to vary simultaneously rather than one at a time. Moreover, the interactions between the factors can be studied.

In this paper, a three-dimensional LSH model of a pitch bearing raceway made in 42CrMo4 steel was built using the finite element software ABAQUS. This model is used to predict the effects of the treatment due to different parameter combinations and study their relative influence on the hardening results. Based on the simulation, according to the uniform experimental design method, a Kriging approximate model is established to predict the results of laser surface quenching.

## 2. Finite Element Simulation of Pitch Bearing Ring

In the laser surface hardening process of the raceway, if the temperature exceeds the phase transition temperature, the phase transition occurs. The hardened depth can be predicted according to the temperature distribution caused by the process. For the pitch bearing raceway, it is made of 42CrMo4 steel and the depth of the hardened layer can be estimated based on the surface temperature if it is higher than the austenitizing temperature (780 °C) and lower than the melting temperature (1450 °C). Therefore, a numerical model and thermal analysis of the raceway was accomplished in order to obtain the temperature fields and evaluate the hardened layer.

### 2.1. Establishment of Thermal Finite Element Model

**Figure 1:**
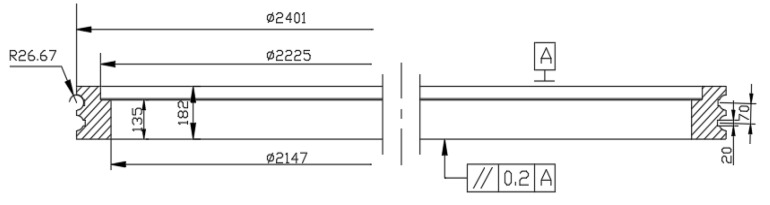
Structure of a pitch bearing inner ring.

**Figure 2:**
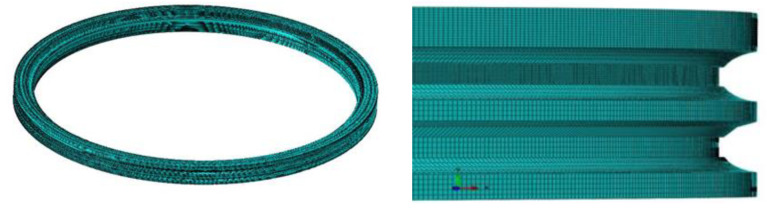
Finite element model and local refinement of the inner ring.

In this study, a three-dimensional thermal finite element model is developed to simulate the distribution of the temperature field according to the bearing raceway parameters in Table 1 and the geometrical structure shown in Figure 1. Due to the complex changes in the actual laser hardening process, simplifying assumptions are made. The pitch bearing raceway is treated by using laser hardening which is a rapid and localized surface processing without component distortion [11,20]. The details of the pitch bearing raceway are simplified by ignoring the bolt hole and inner tooth which have little influence on the surface temperature field distribution. The finite element model of the inner ring structure of the pitch bearing after simplification is shown in Figure 2. The eight-node linear heat-transferred hexahedral element DC3D8 was used. The raceway that needs to be quenched is refined to a size of 0.5 mm, the other zone is 1 mm, and it consists 415,672 elements.

During the simulation process, the value of the temperature at each node is obtained by the transient heat conduction equation which is shown below. The temperature distribution of the object is T (x, y, z, t). Assuming that the inside of the object is isotropic, the temperature distribution should satisfy the basic thermal differential equation:

(1) $$\frac{\partial }{\partial x}\left(\lambda \frac{\partial T}{\partial x}\right)+\frac{\partial }{\partial y}\left(\lambda \frac{\partial T}{\partial y}\right)+\frac{\partial }{\partial z}\left(\lambda \frac{\partial T}{\partial z}\right)=\frac{\partial }{\partial t}\left(\rho C_{p}T\right)$$

where λ is the thermal conductivity of the material (W/m⋅C); ρ is material density ($kg/m^{3}$); $C_{p}$ is specific heat capacity (J/kg⋅C); and t is the time (s). Where T = T (x, y, z, v, P, t) is the temperature field in the material. The temperature fields T is related to the coordinates (x, y, z), the laser processing time t, the laser input power P, and the scanning speed v of the laser beam.

### 2.2. Thermal Properties

During the process, the thermal properties for 42CrMo4 including specific heat capacity and thermal conductivity will change according to heating temperature. Specific heat which expresses a material’s ability to accumulate energy in thermal form with increasing temperatures should be considered in terms of its variation with temperature. Thermal conductivity, which represents the energy transferred per surface unit and time under a temperature gradient of one unit, needs to be considered of its temperature-dependent variation [12].

Because the temperature-dependent material properties for 42CrMo4 are unknown, in this paper, the JMatPro software which can calculate materials properties for multi-component alloys is used to calculate the properties of 42CrMo4 with temperature changes [29]. The specific heat capacity and thermal conductivity of 42CrMo4 within a certain tempering range according to the chemical composition of the material are calculated, and the results are shown in Table 2 [33].

### 2.3. Initial Condition and Boundary Conditions

The temperature at the beginning of computation was uniform at 20 °C. The moving heat sources Gaussian model is used for FEM simulation, considering the convection boundary conditions of thermal analysis. The convection heat transfer coefficient which varies with temperature is shown in Table 3 [33].

## 3. Discussion of Simulation Results

**Figure 3:**
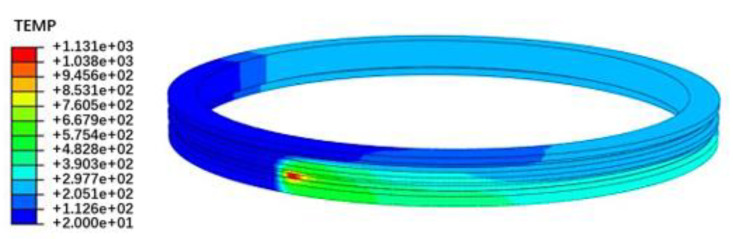
Distribution of temperature field of the raceway.

For example, the following process parameters were chosen: laser power P = 2400 W, laser scanning speed v = 480 mm/min, and spot diameter D = 14 mm. The temperature field distribution of the raceway is shown in Figure 3. As shown, the maximum temperature reaches 1131 °C, which reaches the laser quenching temperature and does not exceed the melting point temperature. The heat is mainly concentrated on the center of the spot. The temperature distribution of the cross section of the raceway was obtained. The hardened depth is estimated based on the node temperature on the section when the temperature is higher than the austenitizing temperature 780 °C and lower than the melting temperature 1450 °C. The multiple points along the depth direction at the center of the spot were selected and used to evaluate the depth taking into account the mesh size. According to the temperature result, the hardened layer depth is predicted to be 1.2 mm based on the method which is thoroughly explained and verified in [33].

**Figure 4:**
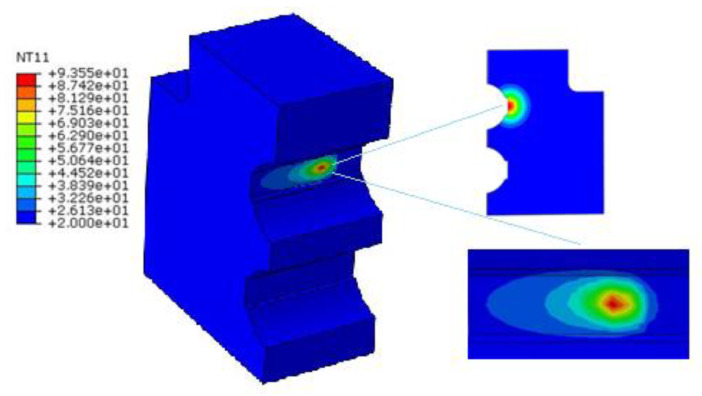
Laser quenching temperature field distribution of pitch bearing raceway.

The results show that the hardened layer depth at the entrance end of the raceway is usually smaller. The depth of the hardened layer in the middle of the raceway and the termination is larger than the entrance positions. For the hardened layer on the cross section, since the temperature in the middle of the spot is higher and the edge temperature is lower, the hardened layer at the center of the laser is deep, and the hardened layer away from the center is shallower, showing a crescent shape, as shown in Figure 4 (hardened area depth and width). However, ideally, it is usually desirable to have a more uniform temperature layer so that a uniform hardened layer can be obtained.

**Figure 5:**
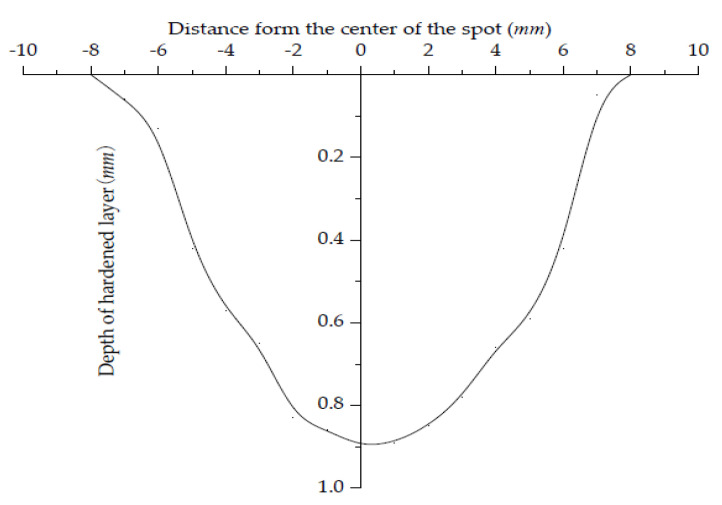
Hardened layer distribution curve.

It can be seen from Figure 5 that the depth of the hardened layer in the center of the quenching zone can be up to 0.98 mm. Far away from the center of the spot, the depth of the hardened layer gradually decreases, and the width of the hardened layer can reach 16 mm.

The temperature in the center area of the laser hardening zone is relatively high, while the edge temperature is relatively low. This is mainly because, during the laser irradiation process, the edge area heats up faster due to the cold action of the substrate. The heat dissipation of the central part is restricted, and the thermal conductivity of the workpiece is higher than that of air, and most of the heat in the central area will be transferred to the depth direction of the inner part. Therefore, the center of the hardened zone is deeper, which is the same as the trend in literature [14,20].

In order to obtain a uniform hardened layer depth of the raceway, a spot shaping treatment can be adopted. The energy distribution at the center of the spot is slightly lower, and the energy distribution at the edges is higher. In addition, the processing method of segmented different process parameters, for example, high-power, low scanning speed at the starting positions, and the relative low-power and higher scanning speed at the middle and ending positions, was adopted.

## 4. Effects of Process Parameters on the Hardened Depth

### 4.1. Effect of Laser Power

**Figure 6:**
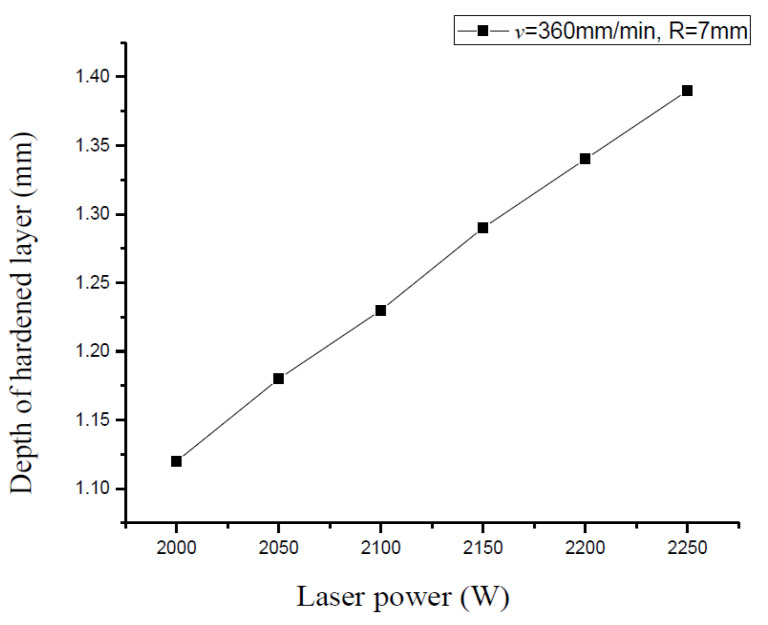
The relationship between different laser power and hardened layer depth.

In order to study the effect of the single process parameter on the depth of the hardened layer, the other two parameters are taken as the constants. The scanning speed v = 360 mm/min, the spot radius R = 7 mm, and the laser power P are 2000, 2050, 2100, 2150, 2200, and 2250 W, respectively. The hardened depth for different laser power is shown in Figure 6.

It can be seen from Figure 6 that with the increase in input power, the hardening layer of the raceway after laser quenching is deeper. Too high laser power will lead to the melting state of the material surface. The surface quality of the material is affected and the hardness may be reduced too.

### 4.2. Effect of Scanning Speed

**Figure 7:**
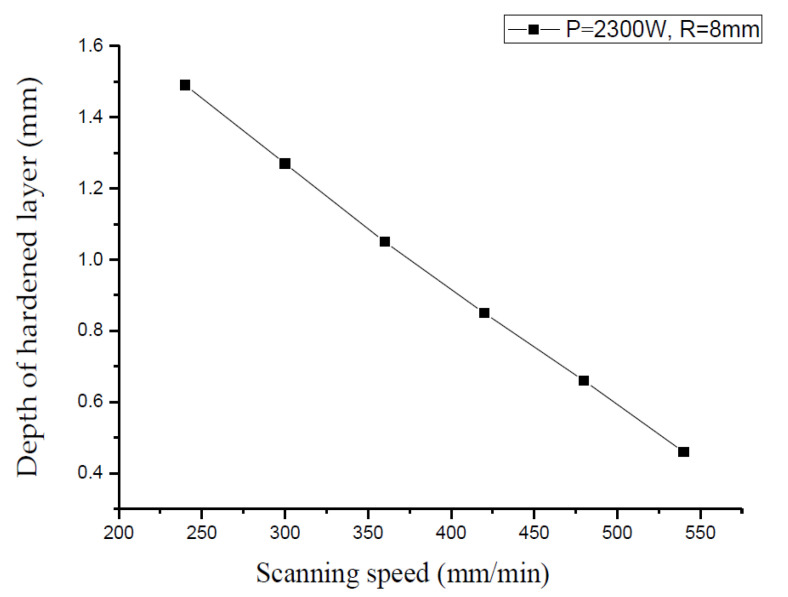
The relationship between different scanning speed and hardened layer depth.

When the laser power P is 2300 W, the spot radius R = 8 mm, and the scanning speed v are 300, 360, 420, 480, and 540 mm/min, respectively, the influence of the scanning speed on the depth of the hardened layer is shown in Figure 7.

It can be seen from Figure 7 that when the laser power and spot diameter are constant, the faster the scanning speed, the smaller the depth of the hardened layer. This is mainly because the laser beam moves too fast and the spot irradiates the workpiece for a shorter time; thus, the metal absorbs less energy. Conversely, the slower the scanning speed, the deeper the hardened layer, but when the scanning speed is too slow, it will cause the surface of the material to melt.

### 4.3. Effect of Spot Size

**Figure 8:**
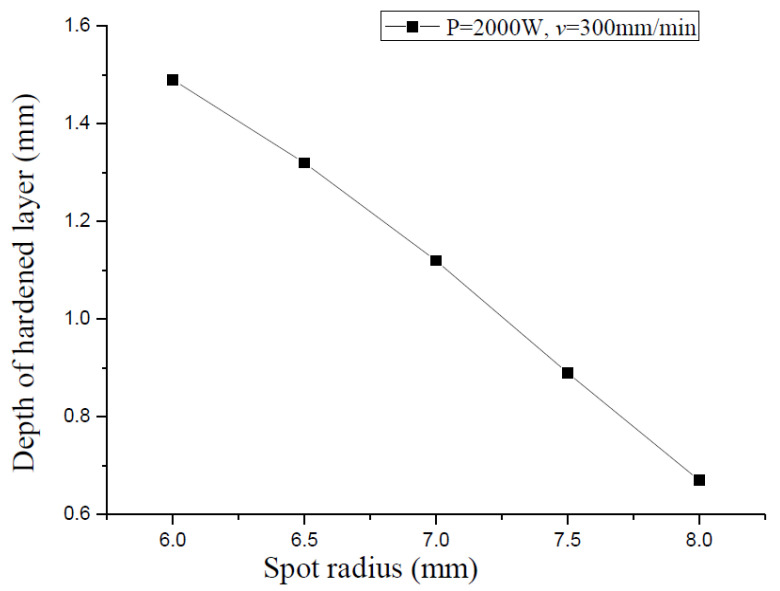
The relationship between different spot radius and hardened layer depth.

When the laser power P is 2000 W, the scanning speed v is 360 mm/min, and the spot radius R are 5, 6, 6.5, 7, 7.5, 8, 8.5, and 9 mm, respectively. The depth of the hardened layer is shown in Figure 8.

It can be seen from Figure 8 that when the laser power and scanning speed are constant, as the diameter of the spot becomes larger, the depth of the hardened layer becomes smaller. This is mainly because the larger the spot diameter, the lower the power density, and thus the shallower the hardened layer. When the laser power is 2000 W, the scanning speed is 360 mm/min and the spot radius is 5 mm. The maximum surface temperature of the material reaches 1886.96 °C, which is much higher than the melting point temperature of the material, and the hardened layer depth cannot be calculated. Therefore, the spot diameter is not as small as possible. When ensuring that the maximum surface temperature is less than the melting point of the material, the smaller the spot diameter can appropriately increase the depth of the hardened layer, but at the same time the width of the laser beam scanning treatment is also correspondingly narrow.

### 4.4. Effect of Power Density

**Figure 9:**
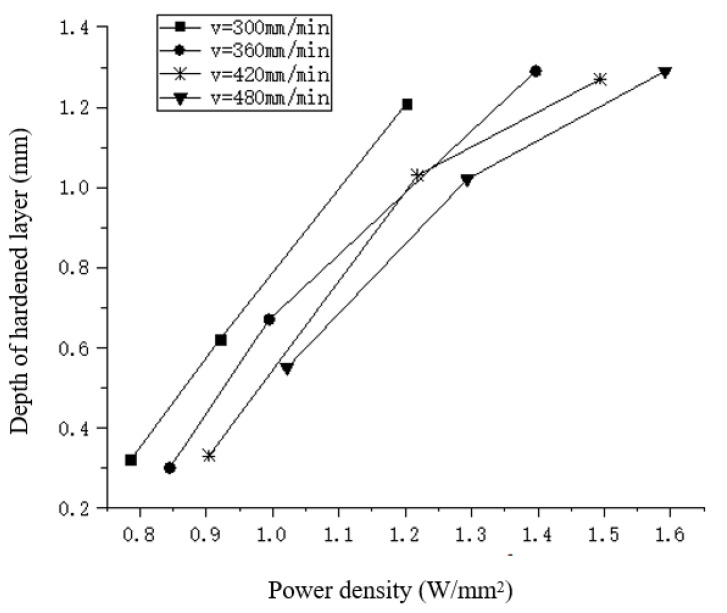
The relationship between different power density and hardened layer depth.

The research results show that the combination of laser process parameters has a high impact on the depth of the hardened layer. The three parameters have a coupling influence on each other. Therefore, the effect of the power density on the depth of the hardened layer is studied. The analysis of the influence of the laser power and spot diameter on the distribution of the hardened layer depth is shown in Figure 9 which shows the relationship between different power density and the depth of the hardened layer.

It can be seen that when the scanning speed is 300 mm/min, 360 mm/min, 420 mm/min, 480 mm/min, respectively, the change trend of the relationship between the power density and the depth of the hardened layer is the same. The higher the power density is, the higher the surface temperature and the deeper the depth obtained. Therefore, the power density directly effects the temperature distribution and the depth during laser quenching.

## 5. Design of Experiments and Construction of Kriging Models

### 5.1. Foundation of Kriging Method

The Kriging interpolation model is also called the best interpolation method of spatial autocovariance [29,30,31]. The Kriging model consists of two parts: the global model and local deviations [29,30,31], which is shown as follows:

(2) $$y\left(x\right)=F\left(\beta ,x\right)+z\left(x\right)=f^{T}\left(x\right)\beta +z\left(x\right)$$

where β is the regression coefficient. $f^{\;}\left(x\right)$ is the global regression model, in the form of a polynomial function. It has no critical influence on the fitting accuracy, and it is often set to a certain constant. $z\left(x\right)$ is a random process with a covariance of $\sigma^{2}$ and a mean of zero. It provides an approximation to simulate the local deviation, that is the local variation of $y\left(x\right)$. $z\left(x\right)$ is normally distributed with a non-zero covariance.

The covariance matrix of interpolation points $x_{i}$ and $x_{j}$ is:

(3) $$Cov\left(x\right)\left[Z\left(x_{i}\right),Z\left(x_{j}\right)\right]=\sigma^{2}R\left(x_{i},x_{j}\right)$$

$R\left(x_{i},x_{j}\right)$ is the spatial correlation equation of two random sample points $x_{i}$ and $x_{j}$. $R\left(x_{i},x_{j}\right)$ determines the accuracy of the simulation process. Gaussian correlation equation is widely used with good calculation efficiency. The form is as follows:

(4) $$\;\;\;R\left(x_{i},x_{j}\right)=\exp\left(-\sum_{k=1}^{n_{d}}\theta_{k}{\left|x_{k}^{i}-x_{k}^{j}\right|}^{2}\right)$$

where $n_{d}$ is the number of design variables, $\theta_{k}$ is the unknown parameter in the related equation.

In order to obtain the above parameter $\theta_{k}$, suppose the response value is $Y={\left[y_{1},y_{2},\cdots ,y_{d}\right]}^{T}$, then the estimated value of the response parameter $\;y\left(x\right)$ at the unknown point x can be expressed as:

(5) $$\hat{y}=C^{T}Y$$

The error obtained by the simulation calculation is:

(6) $$\hat{\;y}-y=C^{T}Y-y=C^{T}\left(F\beta +Z\right)-\left(f^{T}+z\right)=C^{T}Z-z+{\left(F^{T}C-f\right)}^{T}$$

$\mu \left(\hat{y}-y\right)=0$, so the following formula must be satisfied:

(7) $$\;\;F^{T}C-f=0$$

And the mean square error of the error is:

(8) $$\begin{array}{c} \varphi \left(x\right)=E\left[{\left(\hat{y}-y\right)}^{2}\right]=E\left[{\left(C^{T}Z-z\right)}^{2}\right]=E\left(Z^{2}+C^{T}ZZ^{T}C-2C^{T}Zz\right)\\ =\sigma^{2}\left(1+C^{T}RC-2C^{T}r\right) \end{array}$$

(9) $$r\left(x\right)=\left[R\left(x,x^{1}\right),R\left(x,x^{2}\right),\cdots ,R\left(x,x^{n_{d}}\right)\right]$$

where x is the point to be measured, and $r\left(x\right)$ represents the correlation vector between x and the sample point.

The Lagrangian function for the problem of minimizing $\varphi \left(x\right)$ with respect to C and subject to the constraint Equation (7) is:

(10) $$L\left(C,\lambda \right)=\sigma^{2}\left(1+C^{T}RC-2C^{T}r\right)-\lambda^{T}\left(F^{T}C-f\right)$$

The gradient of Equation (10) with respect to C is:

(11) $$L^{\prime }\left(C,\lambda \right)=2\sigma^{2}\left(RC-r\right)-F\lambda$$

From the first-order necessity condition, the following system of equations is obtained:

(12) $$r\left(x\right)\left[\begin{array}{cc} R & F\\ F^{T} & 0 \end{array}\right]\left[\begin{array}{c} C\\ \tilde{\lambda } \end{array}\right]=\left[\begin{array}{c} r\\ f \end{array}\right]$$

The solution is:

(13) $$\tilde{\lambda }=-\frac{\lambda }{2\sigma^{2}}={\left(F^{T}R^{-1}F\right)}^{-1}-\left(F^{T}R^{-1}r-f\right)$$

(14) $$C=R^{-1}\left(r-F\tilde{\lambda }\right)$$

The approximation $\hat{y}$ at the point x to be predicted has the following form:

(15) $$\begin{array}{l} \hat{y}=C^{T}Y=r^{T}R^{-1}y-{\left(F^{T}R^{-1}r-f\right)}^{T}{\left(F^{T}R^{-1}F\right)}^{-1}F^{T}R^{-1}y\\ =r^{T}R^{-1}y-{\left(F^{T}R^{-1}r-f\right)}^{T}\beta^{*}=f^{T}\beta^{*}+r^{T}R^{-1}\left(Y-F\beta^{*}\right) \end{array}$$

Therefore, for each new sample, when the global regression model f and the correlation vector r are determined and the corresponding response value is quickly calculated.

The logarithmic likelihood function of $y\left(x\right)$ is:

(16) $$-\frac{1}{2}\left[\left(n_{d}ln\sigma^{2}+ln\left|R\right|\right)+{\left(Y-F\beta^{*}\right)}^{T}R^{-1}\left(Y-F\beta^{*}\right)/\sigma^{2}\right]$$

The $\;\beta^{*}$ and $\sigma^{2}$ are estimated using the following equation:

(17) $$\beta^{*}={\left(F^{T}R^{-1}F\right)}^{-1}F^{T}R^{-1}y$$

(18) σ2=(y−Fβ*)Ty−Fβ*nd

Substituting $\beta^{*}$ and $\sigma^{2}$ into Equation (16), the following maximization problem is obtained as:

(19) $$\underset{\theta_{k}>0}{max}\left[-\frac{1}{2}\left(n_{d}ln\sigma^{2}+ln\left|R\right|\right)\right]$$

The maximum likelihood estimation of parameter $\theta_{k}$ is obtained by solving the nonlinear unconstrained optimization problem.

After the Kriging model is established, the model can be used to predict the response of new sample points. In this paper, the response sample points are computed by the finite element method according to the above process.

### 5.2. Kriging Approximation Model

The laser power P (X1), scanning speed v (X2), and spot radius R (X3) are selected as input parameters. Two parameters of maximum temperature and depth of hardened layer are selected as the output response to evaluate the hardening effect after laser quenching. The uniform method is adopted for the design of experiments to obtain the relationship between input parameters and output response. The ranges of input parameters are 1850~2600 W, 300~480 mm/min, and 7~9 mm, respectively.

Kriging models are used to construct the complex nonlinear relationship between process parameters and hardening effects. This procedure is run in Matlab software and the function ‘dacefit’ in the DACE toolbox is used. The linear regression model is chosen as the polynomial function because it has no effect on the precision of the model, which means f(x) = 1. The Gaussian correlation equation is selected for its good calculation. Kriging approximate model with two response variables is established by solving the unconstrained optimization problem based on the sample data listed in Table 4. According to the sample points, twelve sets of the obtained sample points and the simulation results of the output response for these sample points are listed in Table 4.

The Kriging approximate model is established based on the above description in detail. Parameters of the unbiased Kriging approximate model were obtained as listed in Table 5.

In order to verify the precision of the Kriging approximate model, random combinations of process parameters are chosen in the sample space. Using the established approximate model, the response of the new sample points is predicted to verify the effectiveness of the model. In the experimental variable space, five groups of process parameters were selected. The Kriging model was used to predict the maximum temperature and hardening depth and the corresponding finite element analysis was completed.

The results of the Kriging model prediction and comparison with finite element analysis are shown in Table 6. For the first and last set of data in Table 6, because the maximum temperature is lower than the phase temperature of 780 °C and higher than the melting point temperature of 1450 °C, respectively, the depth of hardened layer is not considered. It can be seen from the data in the table that the maximum error accuracy of the Kriging model established in this paper is controlled within 10%, and the range of relative error is acceptable. The approximate model can replace the finite element simulation of laser surface hardening, and can be used to predict the temperature value and hardened layer depth under different combinations of process parameters.

### 5.3. Construction of Response Surface

**Figure 10:**
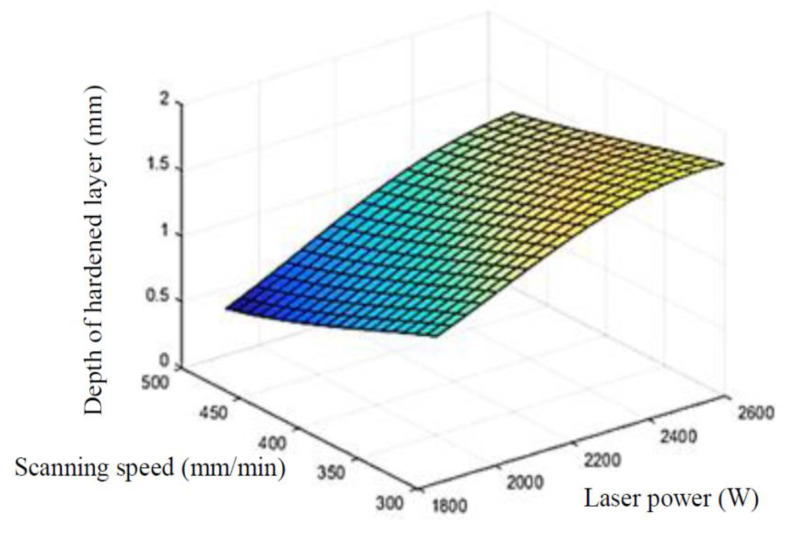
Kriging models for depth of hardened layer of scanning speed and laser power.

**Figure 11:**
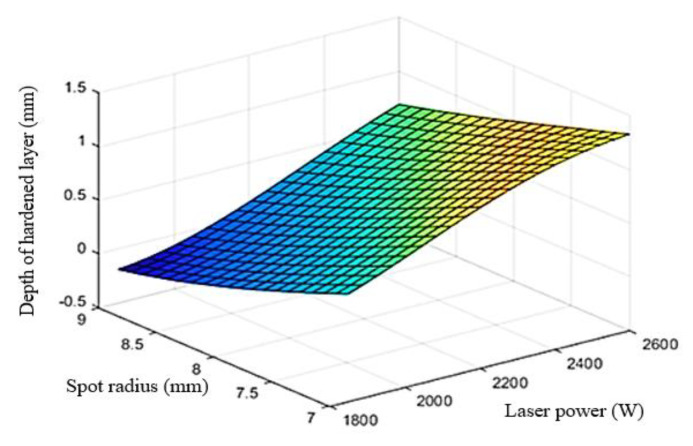
Kriging models for depth of hardened layer of laser power and spot radius.

Based on the Kriging approximation model, the three-dimensional response surface between process parameters and hardened layer depth was drawn. Figure 10 shows the response surface of laser power and scanning speed, Figure 11 shows the response surface of laser power and spot radius. From the response surface of hardened layer depth, laser power, scanning speed, and spot radius after laser quenching, it can be seen that the process parameters are interacted and controlled together. Reasonably increasing laser input power, reducing scanning speed and spot radius can improve the hardened layer depth.

## 6. Conclusions

(1) The three-dimensional finite element model of the large-scale pitch bearing ring was established. The transient temperature field of the laser surface hardening process was studied. The proposed model can provide an approach to analyzing the influence of different process parameters to evaluate the hardened layer. However, in the thermal simulation, the heat flux involved remains unchanged which should be extended so that it would be able to take into account the actual flux parameters in a further and more sophisticated model. The properties of the material as presented in this paper should be verified by means of experiment analysis.
(2) The effects of three process parameters on the depth of the hardened layer were analyzed. The influence of process parameters shows that the laser power has a significant effect on the hardening depth. The scanning speed and spot diameter have less influence on the hardening depth when the other two parameters are constant. The faster the scanning speed and the larger the spot diameter, the shallower the depth of the hardened layer. Comprehensively considering the influence of the three process parameters, choosing a smaller laser power, a lower scanning speed, and a reasonable spot diameter can get a better hardening effect.
(3) A method for constructing a Kriging mathematical model of the laser surface quenching process effect based on the finite element method is proposed, and the hardening layer depth prediction model is established.The constructed Kriging model between process parameters and hardening layer has a small relative error with the simulation results.
(4) The maximum temperature and hardening layer depth can be obtained through the pending process parameters by using the Kriging model, which reduces the experimental cost. This simplifies the complexity of the numerical simulation and is beneficial to be applied to engineering applications.

## Figures and Tables

**Table 1:** Design parameters of pitch bearing raceway.

Design Parameters of Pitch Bearing Raceway	Value
Nominal diameter d	2225 mm
Diameter of center circle of roller Dm	2401 mm
Pitch diameter D	2620 mm
Raceway radius of curvature r	26.67 mm
contact angle α	45°

**Table 2:** Specific heat capacity and thermal conductivity of 42CrMo steel.

Temperature T (°C)	Specific Heat C (J/(kg*°C)	Thermal Conductivity (W/m*°C)
25	460.43	58.682
100	485.32	50.708
200	514.61	48.112
300	570.14	45.689
400	585.73	41.718
500	648.49	38.279
600	707.07	33.943
700	769.82	30.13
800	623.39	32.896
900	548.08	29.756
1000	628.46	29

**Table 3:** Convection heat transfer coefficient of 42CrMo4.

Temperature/°C	Convection Heat Transfer Coefficient/ W/(m^2^*°C)
0	2.5
25	2.66
60	3.5
100	5
200	13.85
300	10
400	3

**Table 4:** Uniform experimental design and response parameters.

Experiment No.	Process Parameters			Response Parameters	
	P (W)	v (mm/min)	R (mm)	Maximum Temperature (°C)	Hardened Depth (mm)
1	1850	300	7	1199.04	1.21
2	2000	360	8	954.50	0.67
3	2150	360	9	843.33	0.30
4	2300	420	9	854.013	0.33
5	2450	480	7	1258.41	1.29
6	2600	480	8	1089.63	1.02
7	1850	300	8	937.82	0.62
8	2000	300	9	849.65	0.32
9	2150	360	7	1249.49	1.29
10	2300	420	7	1245.60	1.27
11	2450	420	8	1092.60	1.03
12	2600	480	9	912.13	0.55

**Table 5:** Parameters of unbiased Kriging model.

	$\theta_{1}$	$\theta_{2}$	$\theta_{3}$
Temp	0.2726	0.1000	0.1072
Depth	0.1393	0.0152	0.1838

**Table 6:** Comparison of Kriging model prediction results and finite element results.

Test Sample Variables			Maximum Temperature (°C)		Error	Depth of Hardened Layer (mm)		Error
$X_{1}$	$X_{2}$	$X_{3}$	FEA	Kriging		FEA	Kriging	
2000	360	9	772.02	770.54	1.91%	-	-	-
2150	300	8	1107.01	1128.60	1.95%	1.09	1.00	8.26%
2200	360	7	1281.04	1305.27	1.89%	1.34	1.35	0.75%
2250	360	9	891.08	890.44	0.07%	0.48	0.45	6.25%
2300	420	6	1557.47	1597.87	2.59%	-	-	-

## Data Availability

Not applicable.
